# Neuronal Progenitors Suffer Genotoxic Stress in the *Drosophila* Clock Mutant *per^0^*

**DOI:** 10.3390/cells13231944

**Published:** 2024-11-23

**Authors:** Nunzia Colonna Romano, Marcella Marchetti, Anna Marangoni, Laura Leo, Diletta Retrosi, Ezio Rosato, Laura Fanti

**Affiliations:** 1 Department of Biology and Biotechnology “Charles Darwin”, Sapienza University of Rome, 00185 Rome, Italy; nunzia.colonna16@gmail.com (N.C.R.); marcella.marchetti@uniroma1.it (M.M.); marangoni.1759731@studenti.uniroma1.it (A.M.); laura.leo@opbg.net (L.L.); retrosi.1856748@studenti.uniroma1.it (D.R.); 2 Neurogenetics Group, Department of Genetics, Genomics & Cancer Sciences, University of Leicester, Leicester LE1 7RH, UK; 3 RNA Editing Lab., Onco-Haematology Department, Genetics and Epigenetics of Paediatric Cancers, Bambino Gesù Children Hospital, IRCCS, 00179 Rome, Italy; 4 Istituto Pasteur Italia, Sapienza University of Rome, 00185 Rome, Italy

**Keywords:** clock, *period*, *Drosophila*, brain, development, genotoxic stress, genome stability

## Abstract

The physiological role and the molecular architecture of the circadian clock in fully developed organisms are well established. Yet, we have a limited understanding of the function of the clock during ontogenesis. We have used a null mutant (*per^0^*) of the clock gene *period* (*per*) in *Drosophila melanogaster* to ask whether PER may play a role during normal brain development. In third-instar larvae, we have observed that the absence of functional *per* results in increased genotoxic stress compared to wild-type controls. We have detected increased double-strand DNA breaks in the central nervous system and chromosome aberrations in dividing neuronal precursor cells. We have demonstrated that reactive oxygen species (ROS) are causal to the genotoxic effect and that expression of PER in glia is necessary and sufficient to suppress such a phenotype. Finally, we have shown that the absence of PER may result in less condensed chromatin, which contributes to DNA damage.

## 1. Introduction

The circadian clock is a timing mechanism that tunes biochemistry and physiology to the environment. In *Drosophila melanogaster* flies, the clock revolves around the expression of two genes, *period* (*per*) and *timeless* (*tim*), that are regulated by their own protein products. Rhythmic expression starts during the day with the transcription of *per* and *tim* by CLOCK/CYCLE (CLK/CYC), a heterodimeric transcription factor. After synthesis, PER and TIM undergo a maturation process that begins in the early evening comprising dimerization, progressive post-translational modifications, and accumulation. Late at night, the two proteins become nuclear and competent inhibitors of CLK/CYC. However, PER and TIM modifications trigger their degradation, which releases the inhibition on CLK/CYC. Thus, during the day, *per* and *tim* transcription starts again, beginning a new cycle [1]. Mammals have a circadian clock also, which is fundamentally similar to the one in *Drosophila* [2]. Importantly, CLK/CYC and the homologous CLK/BMAL1 in mammals, directly and indirectly, control the expression of a large part of the genome [3,4,5,6,7]. This suggests that the clock and/or its constituents may be important regulators of chromatin structure [8,9,10,11,12].

There is evidence that components of the circadian clock are expressed during early ontogenesis, but the role that they, or the clock as a process, play in development is unclear [13,14,15,16]. There is a well-known interdependence between the circadian clock and metabolism in fully developed organisms [17]. Interestingly, metabolic reprogramming is both cause and effect of changes in differentiation status during development [18]. This suggests that the circadian clock, or its parts, may be involved in the developmental programme of the organism.

In this study, we explore the role PER may have in the development of the nervous system in *Drosophila*. Mutants *per*-null (*per^0^*) grow to adulthood, are fertile, and do not show gross morphological abnormalities. Nevertheless, *per^0^* flies have neurological defects as shown by alterations in circadian rhythms, memory formation and sleep architecture [19]. Additionally, their brain exhibits mild anatomical defects such as an irregular location of a group of neuroendocrine cells, loss of some dopaminergic neurons and an abnormal arborization pattern of a cluster of clock neurons [20,21,22,23]. Overall, these observations suggest that PER may be required during development for the correct assembly of neuronal circuits. Furthermore, as *per^0^* flies have metabolic defects such as reduced mitochondrial function, increased sensitivity to reactive oxygen species (ROS) and shorter lifespan, there may be a link between metabolic and neurological dysfunctions [24,25,26,27,28,29,30].

Our working hypothesis is that lack of PER, via abnormal metabolism, may cause genotoxic stress impacting the developmental programme of the nervous system. Third-instar larvae constitute an informative model since the presence of hundreds of mitotic cells allows us to measure, in addition to DNA damage, chromosomal aberrations in dividing neuronal precursors.

In flies, the formation of the central nervous system (CNS) proceeds through two ontogenetic phases. During early embryogenesis, neuronal stem cells called neuroblasts (NBs) delaminate from the embryonic neuroectoderm, change shape and start dividing. The transition from embryonic to larval development results in the NBs becoming quiescent (with some exceptions). The first larval stage marks the beginning of feeding. The availability of nutrients triggers the reactivation of the cell cycle in NBs. In each brain lobe (BL) there are approximately 100 type I NBs (NBs I). At each division, they generate an NB I and a ganglion mother cell (GMC) that divides once to produce neurons and/or glia. NBs II are much fewer, eight per BL, but undergo a remarkable amplification of their lineages. At every division, an NB II generates another NB II and an intermediate neural progenitor cell (INP). The INP is initially immature, but after maturation it divides 5–6 times, each producing one proliferating INP and one GMC that divides once to give rise to neurons/glia [31,32]. The ventral nerve cord (VNC) contains only NBs I. Thus, in the CNS of third-instar larvae, there are hundreds of mitotic cells at any one time, which is ideal for assessing chromosome integrity in neuronal precursors (Figure S1).

In this report, we use third-instar larvae to show that lack of PER results in DNA breaks and in a high frequency of chromosome aberrations in dividing neuronal precursor cells. These genotoxic effects correlate with a rise in ROS levels. Additionally, we provide evidence that PER expression in glia is necessary and sufficient to avoid chromosome damage. Finally, we establish a link between PER-dependent genotoxic phenotypes and defects in chromatin architecture. We suggest that PER, either on its own or as part of the clock, controls chromatin states by regulating the metabolic programme of the cells and that such a regulation is important for normal development.

## 2. Materials and Methods

### 2.1. Drosophila Strains and Maintenance

Flies were maintained at 25 °C in a 12h–12h light–dark (LD) cycle on maize/glucose/yeast/agar (6.3/6.9/4.4/0.5%) medium using propionic acid (Merck Life Science, Milan, Italy) as a mold inhibitor.

We used the following stocks: *Canton-S* (*CS*), *per^0^* (in *CS* background), yw; *per-GAL4*, and *SG^10^*; *tim^0^* ([33,34,35], from Jeff Hall, Brandeis University, Waltham, MA, USA); *per^0^*; *UAS*-*per16* ([36], from François Rouyer, NeuroPSI–Paris-Saclay Institute of Neuroscience, Saclay, France); *yw*; *tim-GAL4* ([37], from Patrick Emery, UMass, Boston, MA, USA); *w^1118^*; *repo-GAL4/TM3* (#7415, Bloomington *Drosophila* Stock Center, Bloomington, IN, USA); *w^cs^*; *UAS-gRNAacp/CyO*; *UAS-CAS9/TM3* and *w*^cs^; *UAS-gRNAper/CyO*; *UAS-CAS9/TM3* ([38], from Mimi Shirasu-Hiza, Columbia University, New York, NY, USA); *w^1118^*; *dpnEE-Gal4/CyO*; *TM3/TM6* ([39], from Tzumin Lee, Janelia Research Campus, Ashburn, VA, USA); *w^1118^*; *UAS-AOX* ([40], from Howard T. Jacobs, Institute of Medical Technology and Tampere University Hospital, Tampere, Finland); *UAS-Su(var)3-9GFP*/*T(2;3)TSTL* and *w^1118^; UAS-Hp1/CyO* (from Lucia Piacentini, Sapienza University of Rome, Rome, Italy).

### 2.2. Antioxidant Feeding

Pure vitamin C was diluted in water (H_2_O) and added to the standard feeding medium to a final concentration of 40 mM [41]. In vehicle-only controls the same amount of water was added to the medium. Adult flies were transferred to fresh tubes every 2–3 days.

### 2.3. Immuno-Staining and Confocal Microscopy

For whole-mount immunolabelling, the CNS of third-instar larvae were dissected in cold phosphate-buffered saline (PBS) and fixed in 4% paraformaldehyde (PFA) in PBS for 30 min at room temperature (RT). Samples were washed three times in PBS with 0.5% Triton X-100 (0.5% PBS-Tx) for 15 min at RT. Then, they were blocked with 10% normal goat serum in 0.5% PBS-Tx for 1 h at RT and immunolabelled with primary antibodies (diluted in fresh blocking solution) at 4 °C overnight. Samples were washed three times in 0.5% PBS-Tx for 15 min at RT and incubated with secondary antibodies (diluted in 0.5% PBS-Tx) for 3.5 h at RT in the dark. Samples were mounted on slides with an antifade medium (3% propyl gallate, 80% glycerol, 20% 1xPBS, pH 8.5). Five to ten brains were scored for each experiment. Observations were performed either on an Olympus FV1000 (Olympus UK & Ireland, Southend-on-Sea, UK) or on a Zeiss LSM 780 (Zeiss Italia, Milan, Italy) confocal microscope. Microscope, lasers, filters, and all other settings remained constant within each independent experiment. Images were processed using FIJI (release 1.54f) [42].

### 2.4. Antibodies

Primary: chicken α-GFP (1:1000, #ab13970, Abcam, Cambridge, UK), mouse monoclonal α-LacZ (1:1000, #Z3781, Promega, Madison, WI, USA), mouse monoclonal a-γH2Av (1:10, #UNC93-5.2.1, DSHB, Iowa City, IA, USA), mouse monoclonal anti-Repo (1:15, #8D12, DSHB, Iowa City, IA, USA), mouse monoclonal anti-Prospero (1:10, #MR1A, DHSB, Iowa City, IA, USA), rabbit a-PER c-300, (1:50; Santa Cruz Biotech, Dallas, CA, USA).

Secondary: goat anti-mouse AlexaFluor568, (1:1000, #11004, Invitrogen, Milan, Italy), goat anti-mouse Texas Red (1:400, #A-115-005-044, Jackson ImmunoResearch, Cambridge, UK), goat anti-rabbit Cy2 (1:400, #A-111-035-144, Jackson ImmunoResearch, Cambridge, UK) goat anti-chicken AlexaFluor488 (1:400, #A-32931, Invitrogen, Milan, Italy).

### 2.5. Measurement of Mitochondrial ROS

Central nervous systems (CNSs, each consisting of two brain lobes—BLs—and one ventral nerve cord—VNC) from male third-instar larvae were dissected in cold phosphate-buffered saline (PBS) and incubated with 5 μM of MitoSOX Red (Thermo Fisher Scientific, Cambridge, UK) for 30 min at room temperature (RT). After incubation, CNSs were washed for 3 × 5 min with PBS at RT. Then, they were fixed with 4% PFA for 20 min at RT, washed for 3 × 5 min with PBS and mounted in antifade (3% propyl gallate, 80% glycerol, 20% 1xPBS, pH 8.5). Samples were imaged immediately with an Olympus FV1000 confocal laser scanning microscope. Images were acquired as z-stacks through the entire thickness of the BLs and (separately) the VNC using a 20× UPlanSApo Olympus objective. Total fluorescent intensities for the BLs (the two were averaged for each individual) and the VNC were measured using Fiji (release 1.54f) [42].

### 2.6. Mitotic Chromosome Preparations

Mitotic chromosomes from the CNS were prepared as previously published [43]. Briefly, CNSs were dissected from male third-instar larvae in physiological solution (NaCl 0.7%), transferred to hypotonic solution (sodium citrate 0.5%) for 8 min and then moved to a drop of fixing solution (methanol: acetic acid: water = 5.5:5.5:1) for 30 s. Five CNSs were transferred to five drops of 45% acetic acid on a siliconized coverslip. A non-siliconized slide was lowered on the coverslip, and the ‘sandwich’ was inverted and squashed between two sheets of blotting paper for 1 min. Slides were frozen in liquid nitrogen and the coverslips were ‘flung off’ with a razor blade. The slides were immersed in 100% ethanol for 5 min. Then, they were washed in 1xPBS for 10 min and counterstained with DAPI solution (0.05 μg/mL of 4′, 6-diamidine-2-phenylindole dihydrochloride in 2XSSC, Sigma-Aldrich, St. Louis, MO, USA) for 5 min. Samples were mounted in an antifade medium (2.3% DABCO-1, 4-diazabicyclo[2,2,2]octane-, 20 mM Tris-HCl pH 8, 90% glycerol). The slides (4–8 per experiment) were imaged across all areas on an ellipse epifluorescence microscope (E1000 Nikon, Tokyo, Japan) equipped with a cooled CoolSnap CCD camera (Photometrics, Tucson, AZ, USA). Images were processed using Adobe Photoshop CS6.

### 2.7. Immunofluorescence on CNS-Squash Preparations

We followed a previously published protocol [43]. Briefly, CNSs were dissected from male third-instar larvae in physiological solution (NaCl 0.7%), transferred to a hypotonic solution (sodium citrate 0.5%) for 8 min, and then fixed in 45% acid acetic and 2% formaldehyde for 10 min before being squashed (five per coverslip) for 1 min in the same solution. Slides were frozen in liquid nitrogen and the coverslips were removed. Slides were transferred to 1xPBS for 5 min, permeabilized in 1% PBS-Tx for 10 min, and blocked in 1xPBS and 1% BSA for 30 min before incubation with mouse a-γ-H2AV antibodies (DSHB #UNC93-5.2.1) diluted 1:5 in 1xPBS and 1% BSA. Incubation with the primary antibodies was carried out at room temperature for 1 h and then at 4 °C overnight. Samples were washed for 3 × 5 min in 1xPBS. The secondary antibodies, goat anti-mouse Cy3 (Jackson ImmunoResearch, Cambridge, UK), were diluted 1:400 in 1xPBS and 1% BSA and incubated at room temperature for 2 h. Samples were washed for 3 × 5 min in 1xPBS, counterstained with DAPI solution (0.05 μg/mL of 4′, 6-diamidine-2-phenylindole dihydrochloride in 2XSSC) for 5 min and then mounted in Vectashield H-1000 (Vector Laboratories, Newark, CA, USA). The slides (4–8 per experiment) were imaged across all areas using an ellipse epifluorescence microscope (E1000 Nikon, Tokyo, Japan) equipped with a cooled CoolSnap CCD camera (Photometrics, Tucson, AZ, USA). The exposure time was kept constant across all samples. The two fluorescent signals (from DAPI and Cy3), were recorded separately as grayscale digital images. Images were pseudo-coloured and merged using Adobe Photoshop CS6.

### 2.8. Mitochondrial Oxygen Consumption Measurements

High-resolution respirometry measures were performed with an Oroboros Oxygraph-2k (Oroboros Instruments, Innsbruck, Austria). Mitochondrial leak (L), oxidative phosphorylation (OXPHOS) and electron transport system (ETS) capacities were quantified using a previously described substrate–uncoupler–inhibitor titration (SUIT) protocol [44], with the following modifications. Only males, *per*^+^ and *per^0^*, were used for the analyses. The two genotypes were obtained, respectively, by crossing female *CS* to males *per^0^* and vice versa. For each sample, ten 3–5 day old flies (or ten third-instar larvae) from the same genotype, were homogenized together in 800 μL of respiration buffer MiR05 (0.5 mM EGTA, 3 mM MgCl_2_, 60 mM K-lactobionate, 20 mM taurine, 10 mM KH_2_PO_4_, 2 0 mM HEPES, 110 mM sucrose, and 1 g/L BSA, pH 7.1). When testing the two chambers of the oxygraph, each containing 2 mL of MiR05, were loaded with 80 μL of homogenate, one from a *per*^+^, the other from a *per^0^* sample. The chambers were extensively washed between tests, inverting the loading at each cycle.

### 2.9. Statistics and Reproducibility

Data have been plotted and analysed using Graphpad Prism 9.5.1 (La Jolla, CA, USA). We employed Chi-square, Fisher’s exact test, Shapiro–Wilk test, Kolmogorov–Smirnov test, Mann–Whitney test, and two-way ANOVA with Tukey’s post hoc analyses, as appropriate. All statistical tests used were two-tailed. Sample sizes are indicated in figures and/or legends.

## 3. Results

### 3.1. per^0^ Mutants Are Subject to a High ROS Burden

**Figure 1:**
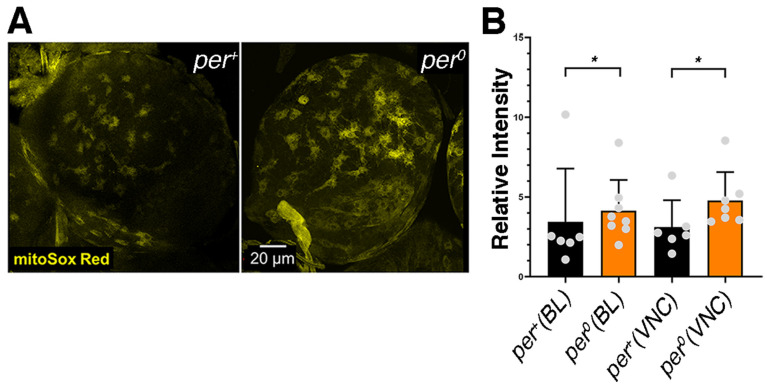

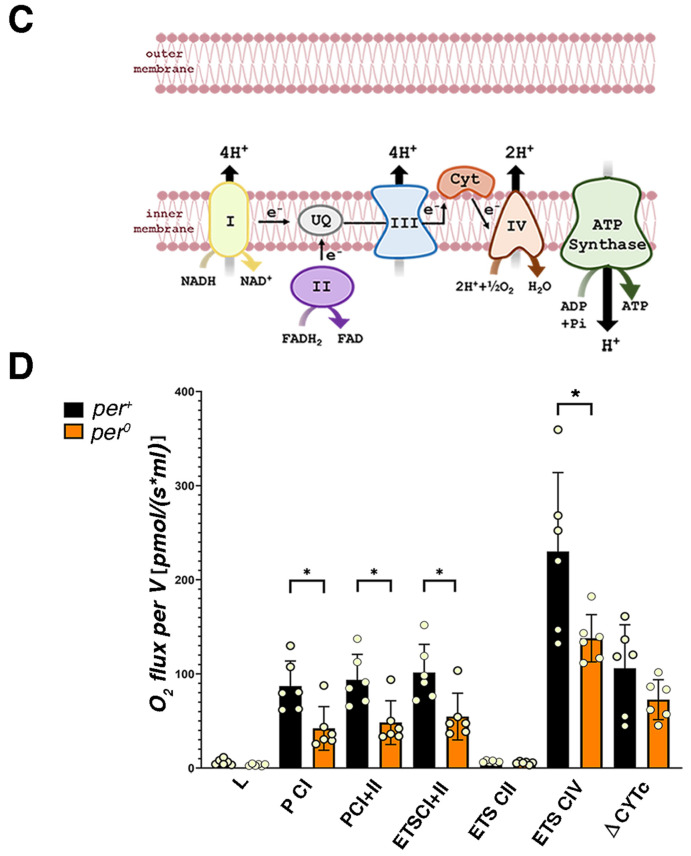
High levels of ROS and mitochondrial respiration defects in *per^0^* mutants. (**A**) Brain lobes stained with mitoSox Red in *per*^+^ and *per^0^* third-instar larvae. Confocal maximum intensity projections. Size bar = 20 μm. ZT = 2. (**B**) Quantification of mitoSox Red signal in *per*^+^ and *per^0^*. Brain lobes (Kolmogorov–Smirnov test, * *p* = 0.042) and ventral cords (Kolmogorov–Smirnov test, * *p* = 0.015) were compared separately. Points show individual samples. Error bars = SD (standard deviation). ZT = 2. (**C**) Cartoon of the mitochondrial complexes involved in oxidative phosphorylation (OXPHOS). (**D**) High-resolution respirometry (oxygen flux per volume [pmol·s^−1^·mL^−1^]) in 3–5 day old *per*^+^ and *per^0^* flies. OXPHOS capacity related to complex I (P CI; Mann–Whitney, * *p* = 0.026) and complex I plus II (P CI + II; Mann–Whitney, * *p* = 0.026) were reduced in *per^0^* compared to *per*^+^ controls. Additionally, *per^0^* showed reduced electron transfer capacity through complex I plus II (ETS CI + II; Mann–Whitney, * *p* = 0.026), and through complex IV (ETS CIV; Mann–Whitney, * *p* = 0.041) but not complex II alone (ETS CII; Mann–Whitney, *p* = 0.132). There were no differences in mitochondrial leak (L; Mann–Whitney, *p* = 0.093) and in mitochondrial integrity (ΔCYTc; Mann–Whitney, *p* = 0.310) between the two genotypes. Points show individual samples. Error bars = SD. ZT = 1. All samples were males obtained by reciprocal crossing (♀ *CS* × ♂ *per^0^* and vice versa).

Previous reports have shown that *per^0^* flies have abnormal metabolism and are sensitive to ROS [21,27,28,29]. We used MitoSOX Red, a fluorogenic superoxide indicator dye that is targeted to the mitochondria, to measure ROS levels in the CNS of larvae (at ZT2, *Zeitgeber* Time 2, corresponding to 2 h after lights on). We detected higher levels of fluorescence in *per^0^* compared to *per*^+^, indicating that the mutant is subject to a higher ROS burden than the control (Figure 1A,B). We used high-resolution respirometry (Oroboros oxygraph) on whole larva extracts (at ZT1) to identify defects in the mitochondrial complexes and the electron transport chain (Figure 1C). Surprisingly, we did not uncover any difference in respiration between *per^0^* and *per*^+^ larvae (Figure S2). We do not have a clear explanation for this finding. Thus, we measured respiration in adult fly extracts (at ZT1). We detected a reduction in the oxidative phosphorylation (OXPHOS) capacity related to complex I and complex I plus II in *per^0^* compared to *per*^+^ controls. Additionally, *per^0^* showed reduced electron transfer capacity through complex I plus II and through complex IV (Figure 1D). A decrease in electron transfer capacity may cause over-reduction in the ubiquinone pool and the formation of superoxide radicals. We suggest that mitochondria may be defective in *per^0^* larvae also and generate high levels of ROS. In summary, we have identified significant respiration defects in *per^0^* flies that may explain the observed ROS increase in larvae.

### 3.2. The CNS of per^0^ Larvae Shows DNA Damage and Chromosome Aberrations

**Figure 2:**
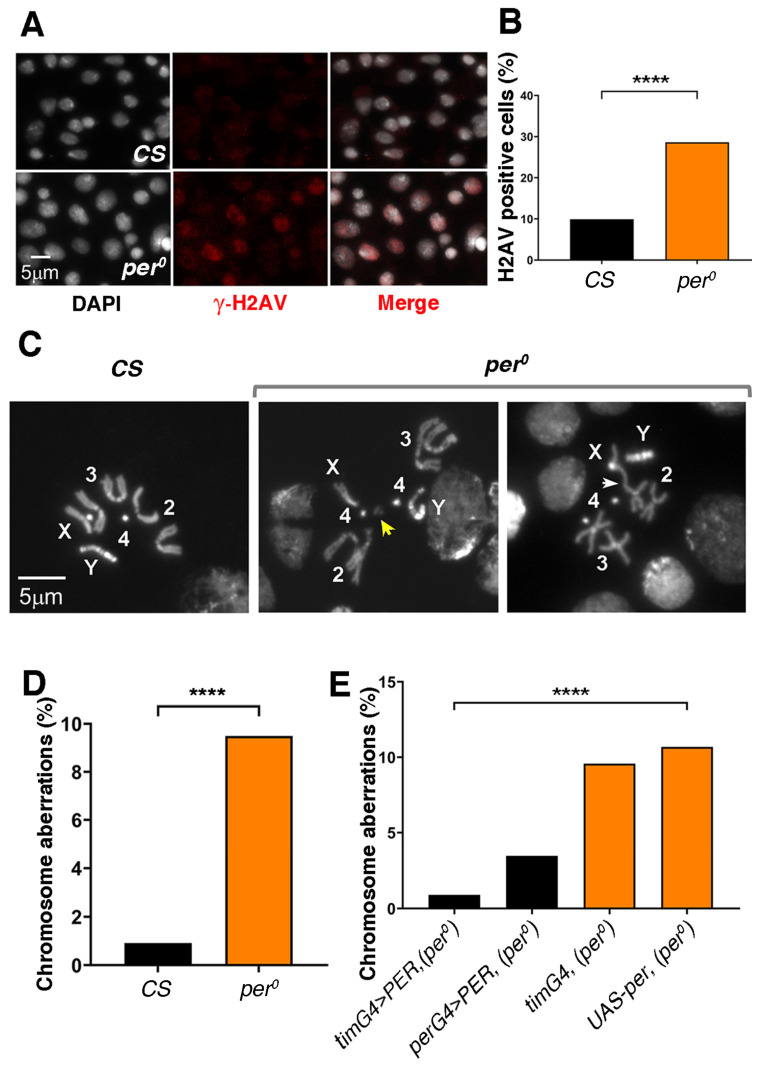
DNA damage and chromosome aberrations in *per^0^* mutants. (**A**) Anti-γ-H2AV immune labelling (red) in CNS-squash preparations from third-instar male larvae. DNA is stained with DAPI (white). Size bar = 5 μm. ZT = 1. (**B**) Proportion of cells showing anti-γ-H2AV immune signal in CNS-squash preparations from *CS* and *per^0^* third-instar male larvae. Cells were considered ‘H2AV positive’ when the relative intensity of the anti-γ-H2AV immune signal in the nucleus [(signal-background)/background] was equal to or more than 1.5. The DAPI signal was used to identify nuclei. Fisher’s exact test, **** *p* < 0.0001. Total number of cells, *n* = 1323, 985, respectively. ZT = 1. (**C**) Mitotic metaphases in CNS-squash preparations from third-instar larvae. Left, *CS*, showing a normal metaphase. Right, chromosome aberrations in *per^0^*. The yellow arrow indicates a chromosome fragment (break). The white arrow points to a fusion. Numbers 2, 3, 4 identify the autosomes. X, Y label the sex chromosomes. Size bar = 5 μm. ZT = 2. (**D**) Frequency of chromosome aberrations [(abnormal metaphases/total metaphases) × 100] in *CS* and *per^0^*. Fisher’s exact test, **** *p* < 0.0001. ZT = 2. Total number of metaphases scored, N = 436, 527, respectively. (**E**) PER overexpression rescues chromosome aberrations in *per^0^*. The overexpression of PER using the pan-circadian *per-GAL4* (*perG4* > PER, *per^0^*) and *tim-GAL4* (*timG4* > PER, *per^0^*) drivers drastically reduced the frequency of aberrations in an otherwise *per^0^* background. Chi-square = 49.50, df = 3, **** *p* < 0.0001. Total number of metaphases scored (from left to right), *n* = 337, 463, 345, 1021. ZT = 2.

Cells exposed to high levels of ROS are prone to oxidative damage, which includes double-strand DNA (dsDNA) breaks [45]. Phosphorylation of histone variant H2AV at serine 137 (referred to as γ-H2AV) marks the recognition of dsDNA breaks and the promotion of repair [46]. Thus, we asked whether *per^0^* larvae may show higher anti-γ-H2AV immune reactivity than *Canton-S* (*CS*) wild-type controls. We produced CNS-squash preparations (at ZT 2), and we labelled them with anti-γ-H2AV (Figure 2A). As expected, the number of immune positive cells was higher in *per^0^* than in *CS* (Figure 2B). Notably, we obtained similar results using whole-mount preparations and larvae that had a more homogenous genetic background. For the latter, we exploited the fact that the *per* locus is X-linked; thus, we selected the male progeny of reciprocal crosses [♀ *per^0^* × ♂ *CS* and vice versa]. These results are illustrated in the following section.

dsDNA breaks may lead to chromosome aberrations (breaks, fusions, de-condensation or translocations, see Figure 2C for an example). We calculated the proportion of mitotic metaphases showing chromosome aberrations in another set of CNS-squash preparations (at ZT2). Indeed, we observed a significantly higher proportion of aberrations in *per^0^* than *CS* larvae (Figure 2D). Then, we tested (at ZT2) whether overexpressing PER (by inducing transcription of *UAS-per*) in *per^0^* larvae, using *per-GAL4* or *tim-GAL4* as a driver, could rescue the high rate of chromosome defects. Larvae *per^0^* expressing either *per-GAL4* > PER or *tim-GAL4* > PER showed wild-type levels of chromosome aberrations (Figure 2E). Since co-expression of *per* and *tim* is one of the defining characteristics of circadian cells, a testable hypothesis is that a defective clock, rather than the specific lack of PER, may be the cause of chromosome damage.

### 3.3. Buffering of ROS Rescues DNA Damage and Chromosome Aberrations in per^0^

**Figure 3:**
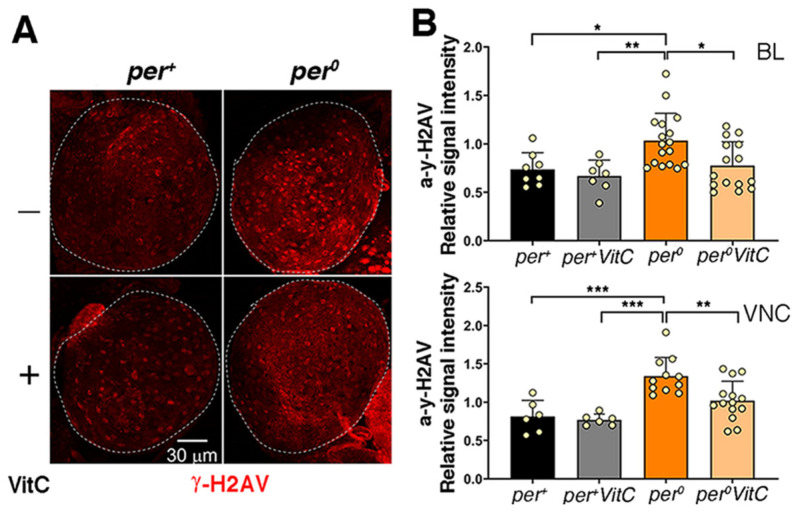

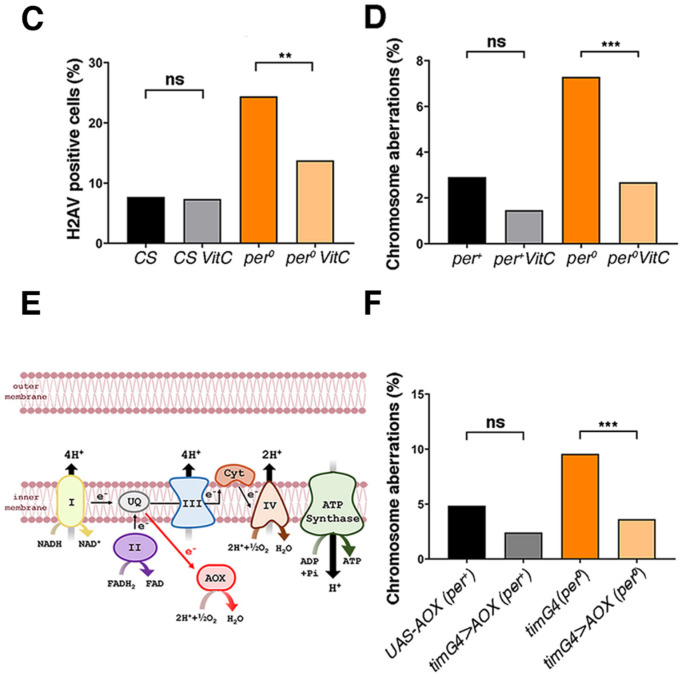
ROS buffering and reduction decrease DNA damage and chromosome aberrations in *per^0^*. Treatment with vitamin C (VitC, 40 mM in standard medium, from embryo) reduced anti-γ-H2AV immune labelling (**A**–**C**) and chromosome aberrations (**D**) in *per^0^* third-instar larva males. (**A**) Brain lobes (BLs) from third-instar *per*^+^ and *per^0^* male larvae obtained by reciprocal crossing (♀ *CS* × ♂ *per^0^* and vice versa); anti-γ-H2AV on the whole mount. Confocal maximum intensity projections. Dashed lines outline the BLs. Size bar = 30 μm. ZT = 2. (**B**) Quantification of anti-γ-H2AV immune fluorescence intensity [relative signal intensity = (signal-background)/background] in whole-mount CNS from third-instar *per*^+^ and *per^0^* male larvae as above. Normal distribution of data was confirmed with the Shapiro–Wilk test. Top, BL (one per individual). Two-way ANOVA, Genotype (F_1, 43_ = 7.318, *p* = 0.0097), VitC treatment (F_1, 43_ = 4.732, *p* = 0.0352), Genotype × VitC treatment (F_1, 43_ = 1.617, *p* = 0.2104). Tukey’s multiple comparisons test, *per*^+^ vs. *per^0^*, * *p* = 0.0282; *per*^+^ VitC vs. *per^0^*, ** *p* = 0.0075; *per^0^* vs. *per^0^* VitC, * *p* = 0.0196. Bottom, VNC. Two-way ANOVA, Genotype (F_1, 33_ = 23.75, *p* < 0.0001), VitC treatment (F_1, 33_ = 5.241, *p* = 0.0286), Genotype × VitC treatment (F_1, 33_ = 3.044, *p* = 0.0903). Tukey’s multiple comparisons test, *per*^+^ vs. *per^0^*, *** *p* = 0.0003; *per*^+^ VitC vs. *per^0^*, *** *p* = 0.0001; *per^0^* vs. *per^0^* VitC, ** *p* = 0.0066. ZT = 1. (**C**) Proportion of anti-γ-H2AV immune positive cells in CNS squash preparations from third-instar male larvae. Cells were considered ‘H2AV positive’ when the relative intensity of the anti-γ-H2AV immune signal in the nucleus [(signal-background)/background] was equal or more than 1.5. The DAPI signal was used to identify nuclei. Treatment with VitC did not affect *CS* (Fisher’s exact test, *p* = 0.9209) but lowered the proportion of anti-γ-H2AV immune labelled cells in *per^0^* (Fisher’s exact test, ** *p* = 0.0011). Total number of cells scored (from left to right), *n* = 912, 775, 295, 298. ZT = 2. (**D**) Proportion of chromosome aberrations [(abnormal metaphases/total metaphases) × 100] in CNS squash preparations from third-instar *per*^+^ and *per^0^* male larvae obtained by reciprocal crossing (♀ *CS* × ♂ *per^0^* and vice versa). Treatment with VitC lowered the proportion of chromosome aberrations. The effect was small in *per*^+^ (Fisher’s exact test, *p* = 0.1886) but highly significant in *per^0^* (Fisher’s exact test, *** *p* < 0.0005). Total number of metaphases scored (from left to right), *n* = 468, 440, 439, 633. ZT = 1. AOX overexpression rescues chromosome aberrations (**E**,**F**). (**E**) Cartoon showing the position of the Alternative Oxidase (AOX) in the electron transport chain. (**F**) The overexpression of AOX using the pan-circadian *tim-GAL4* (*timG4* > AOX) driver reduced the frequency of aberrations. The effect was marginal in *per*^+^ (Fisher’s exact test, *p* = 0.0663) but highly significant in *per^0^* (Fisher’s exact test, *** *p* < 0.0009). Total number of metaphases scored (from left to right), *n* = 397, 387, 456, 412. ZT = 1. Males were obtained by reciprocal crossing [♀ *UAS-AOX* (*per^+^*) × ♂ *tim-GAL4* (*per^0^*) and vice versa].

Vitamin C is a ROS-scavenger without negative side effects in *Drosophila* [41]. Larvae *per^0^* that had developed on medium supplemented with vitamin C (40 mM) showed a significant reduction in dsDNA breaks (anti-γ-H2AV signal) both in whole-mount and in CNS-squash preparations (Figure 3A–C, Figure S3). Chromosome aberrations were likewise reduced (Figure 3D). The expression of *Ciona intestinalis* alternative oxidase (AOX) lessens the production of mitochondrial ROS. AOX avoids the overload of the electron transport chain by accepting electrons directly from the ubiquinone pool and reducing O_2_ into H_2_O [40] (Figure 3E). In *per^0^* larvae, we expressed AOX in all putative clock cells (*tim-GAL4* > AOX), which resulted in the rescue of chromosome aberrations (Figure 3F).

### 3.4. PER Is Expressed in Glia, Including Cortex

**Figure 4:**
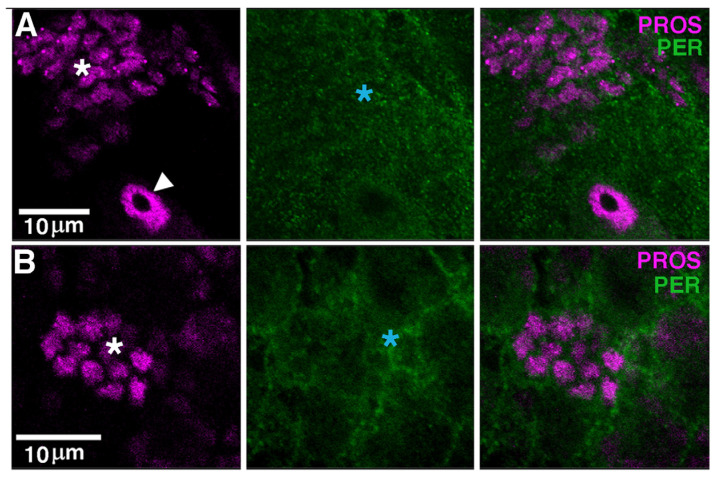

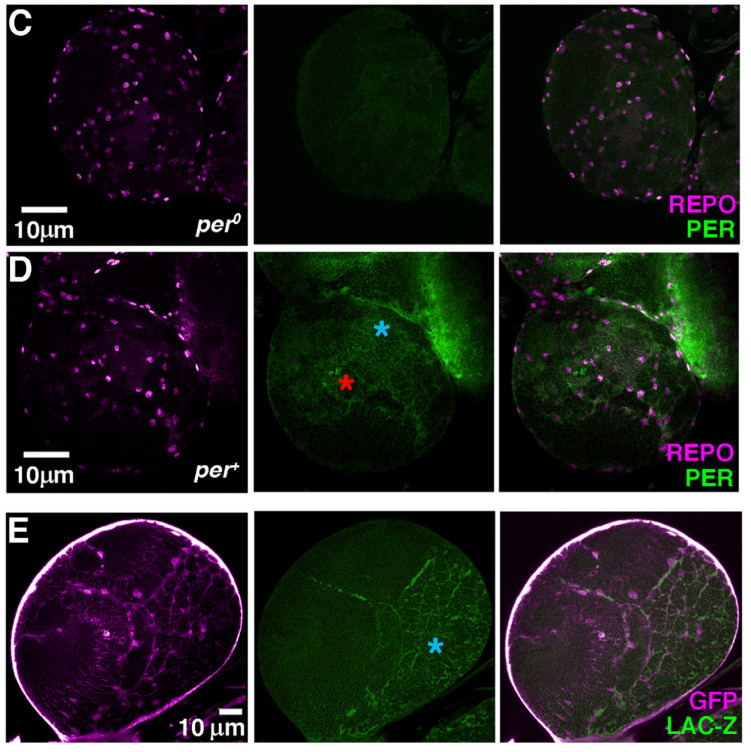
PER is expressed in glia. (**A**,**B**) Optical slices (confocal) showing α-PROS (magenta) and α-PER (green) in brain lobes (BLs) of third-instar larvae. (**A**,**B**) show two independent BLs at slightly different magnification. The white arrowhead shows a type I neuroblast (NB I; note the asymmetric α-PROS cytoplasmic staining) while the white asterisks indicate ganglion mother cells (GMCs; α-PROS nuclear staining). α-PER does not overlap with α-PROS but labels surrounding areas (blue asterisks). Size bars = 10 μm. ZT = 23. (**C**,**D**) Optical slices (confocal) showing α-REPO (magenta) and α-PER (green) in the BLs of third-instar *per^0^* (**C**) and *per*^+^ (**D**) larvae. α-REPO stains the nucleus of glial cells. In *per*^+^, α-PER labels the nucleus of clock neurons (red asterisk) and shows additional weak, diffuse staining (blue asterisk). Size bars = 10 μm. ZT = 23. (**E**) A confocal optical section showing the expression of the PER reporter SG in the BL of a third-instar larva. SG is combined, by crossing, to *repo-GAL4 >* GFP to identify glia through GFP immune reactivity. In the focal plane shown, α-GFP staining (magenta) reveals cortex glia, a type of glia that envelops NBs and their progeny providing niche function. α-LacZ (green) shows overlapping staining (blue asterisk). Size bar = 10 μm. ZT = 2. [Note: magenta and green are pseudo-colours; α-GFP and α-LACZ were imaged in the green—Alexa488—and red—Texas Red—channel, respectively].

In third-instar larvae, there are nine neurons per brain lobe (BL) showing robust and rhythmic PER and TIM expression [33,34,47,48]. These are *bona fide* clock neurons and correspond to clusters recognised in the adult brain. We asked whether PER may be present, additionally, in dividing cells such as NBs and GMCs. The transcription factor PROSPERO (PROS) is asymmetrically distributed in the cytoplasm of NBs I, whereas it becomes nuclear in GMCs [49]. We carried out α-PER and a-PROS immune staining at ZT23, a time when PER is generally nuclear but failed to identify double-labelling in either type of dividing cells (Figure 4A,B). Instead, we observed a weak and diffuse α-PER immune signal around α-PROS immune reactive cells (Figure 4A,B). The NBs and their progeny are enveloped by cortex glia, which provide a ‘niche’ function [49]. Thus, we carried out α-PER and α-REVERSE POLARITY (α-REPO) immune staining (at ZT23); the latter labels the nuclei of all glial cells. Comparing *per^0^* and *per*^+^ larvae further suggested that PER may be expressed, albeit weakly, in the cytoplasm of cortex glial cells (Figure 4C,D). To verify that such a weak α-PER immune reactivity identifies cortex glia, we took advantage of a ‘classic’ PER reporter that accumulates in cells. It consists of a genomic fragment of *per*, including the promoter region and up to the first half of the encoded protein, which is fused in frame to the bacterial *lacZ* gene. The fusion protein, SG, is stable and does not cycle [35]. We employed *repo-GAL4* to express GFP in glia (*SG*, *repo-GAL4* > GFP) and stained larvae with α-GFP and α-LACZ antibodies (at ZT2). To identify cortex glia, we considered the position (underneath the perineurial and subperineurial glia that surround the CNS) and the morphology of the GFP-positive cells. Figure 4E shows that SG is detectable in cortex glia, supporting the notion that PER is expressed in these cells. We note that although Liu et al. (2015) did not observe PER staining in larval glia [48], Kaneko and Hall (2000) identified the expression of a different reporter (*perGAL4* > TAU) in these cells, which provides independent confirmation to our finding [34].

### 3.5. A Non-Cell Autonomous Genotoxic Effect of per^0^

**Figure 5:**
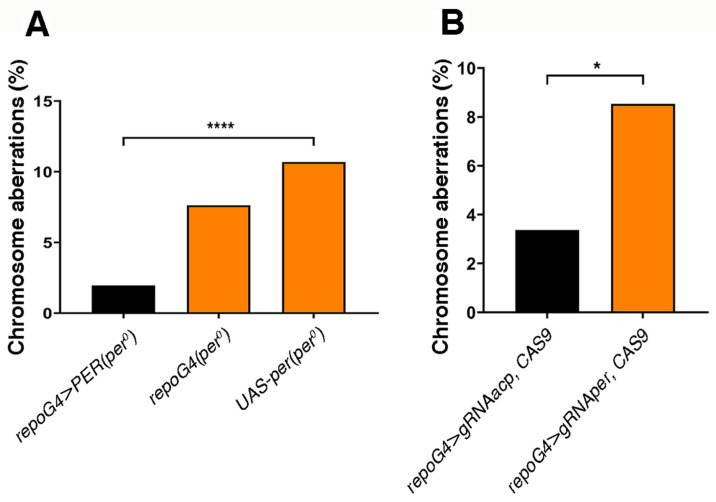
Chromosome aberrations are contingent with the lack of PER expression in glia. (**A**) The overexpression of PER (driving transcription of *UAS-per*) only in the glia of *per^0^* larvae [*repo-GAL4* > PER (*per^0^*)] drastically reduced the frequency of aberrations [(abnormal metaphases/total metaphases) × 100]. Chi-square = 42.21, df = 2, **** *p* < 0.0001. Total number of metaphases scored (from left to right), *n* = 612, 302, 1021. ZT1. (**B**) The knock-out of *per* (with CRISPR/Cas9) only in glia (*repo-GAL4* > *gRNAper*, CAS9) was sufficient to trigger chromosome aberrations. *repo-GAL4* > *gRNAacp*, CAS9 vs. *repo-GAL4* > *gRNAper*, CAS9, Fisher’s exact test, * *p* = 0.0161. Total number of metaphases scored, *n* = 386, 164. ZT = 1.

Since we observed the expression of PER and of its reporter SG in glia, we asked whether chromosome aberrations, which are detected in mitotic cells, are caused by a non-cell-autonomous mechanism. The overexpression of PER in glia, in an otherwise *per^0^* background (*repo-GAL4* > PER, *per^0^*), was sufficient to rescue chromosome aberrations (Figure 5A). We then used a CRISPR/Cas9 approach to carry out the opposite manipulation [38]. We induced somatic mutations in the *per* gene of wild-type larvae by driving the expression of CAS9 and *per* gRNA in glia (*repo-GAL4* > *gRNAper*, CAS9). We observed a significant increase in the proportion of chromosome aberrations compared to the control (*repo-GAL4* > *gRNAacp,* CAS9) that targets CAS9 to *acp98A*, a gene expressed exclusively in the male accessory gland [38] (Figure 5B).

### 3.6. per^0^ Likely Affects the Chromatin Landscape of Neural Progenitor Cells

ROS stress can induce reprogramming of stem cells [50]. One of the possible mechanisms is DNA damage leading to H2AV phosphorylation (i.e., γ-H2AV. This corresponds to the phosphorylation of the homologue histone variant H2AX in mammals) and then activation of POLY (ADP-RIBOSE) POLYMERASE 1 (PARP1), an enzyme that transfers ADP-ribose units from NAD^+^ to target proteins. Such a response promotes the opening of chromatin by de-stabilizing pre-existing protein complexes, which facilitates transcription and genotoxic stress responses but may impact the differentiation status of the cells [51]. Furthermore, in mammalian fibroblasts, the knock-out of the three *Per* genes (*Per1-3*, which correspond to the single *per* in *Drosophila*) causes a reduction in the deposition of H2AZ (an additional homologue histone variant to H2AV) resulting in greater genome accessibility and persistent DNA damage [10]. On this premise, we decided to assess whether there may be relaxation of compacted chromatin in *per^0^*.

**Figure 6:**
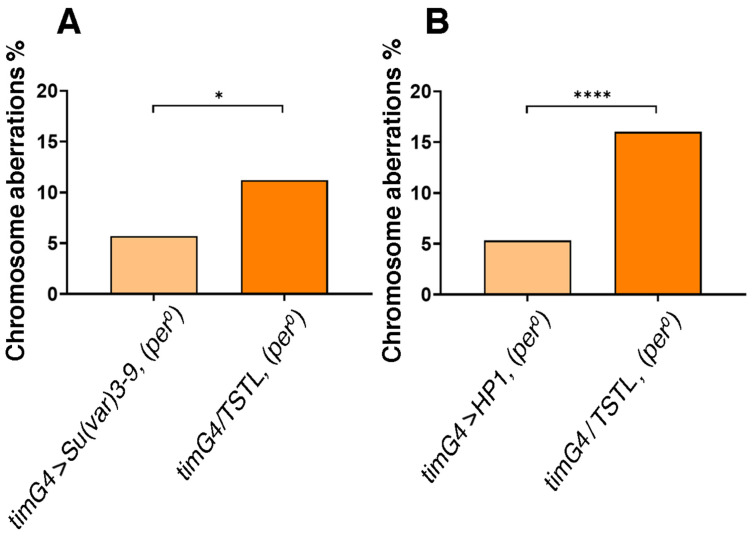
Promoting chromatin compaction reduces chromosome aberrations in *per^0^*. The overexpression of SU(VAR)3-9 (**A**) or HP1 (**B**) using the pan-circadian *tim-GAL4* driver, drastically reduced the frequency of aberrations [abnormal metaphases/total metaphases) × 100] in *per^0^*. (**A**) *tim-GAL4* > SU(VAR)3-9, (*per^0^*) vs. *tim-GAL4/TSTL* (*per^0^*), Fisher’s exact test, * *p* < 0.0129. Total number of metaphases scored, N = 388, 276. ZT = 1. (**B**) *tim-GAL4* > HP1 (*per^0^*) vs. *tim-GAL4/TSTL* (*per^0^*), Fisher’s exact test, **** *p* < 0.0001. Total number of metaphases scored, *n* = 417, 362. ZT = 1. [Note: *TSTL*, *Triplo-Sensitive & Triplo-Lethal* is a chromosomal arrangement that may be used to maintain genetic stability in crosses].

H3K9me3 (tri-methylated histone 3 at Lys9) is an epigenetic mark of constitutive heterochromatin and SUPRESSOR OF VARIEGATION3-9 [SU(VAR)3-9, a histone methyltransferase] and HETEROCHROMATIN PROTEIN 1 (HP1, a structural protein that binds to H3K9me3) are fundamental regulators of its formation and maintenance [52,53]. We used *tim-GAL4* to overexpress SU(VAR)3-9 or HP1 in *per^0^* larvae and we measured chromosome aberrations. Both manipulations reduced the proportion of aberrant metaphases (Figure 6A,B). This suggests that the effect on chromosome integrity we uncovered depends upon an anomalous chromatin landscape in the cells that is caused by lack of PER, or perhaps by a defect of the clock as a whole.

## 4. Discussion

This study reveals that dividing neuronal precursors in third-instar larvae of the circadian mutant *per^0^* show signs of DNA damage. These are in the form of double-strand DNA (dsDNA) breaks (as evinced by high anti-γ-H2AV immune reactivity) and chromosome aberrations (breaks, fusions, translocations, etc.). We provide evidence that such effects are caused by a lack of PER, which results in (i) an increased ROS burden and (ii) changes in the epigenetic landscape of the cells. Intriguingly, PER expression in glia has proven necessary and sufficient to prevent chromosome damage in the dividing cells, revealing that PER is involved in a non-cell autonomous mechanism of protection of the neuronal precursors. Here, we discuss the evidence and speculate about mechanistic implications.

### 4.1. Lack of PER Results in Higher Levels of ROS

Already previous reports had highlighted metabolic deficiencies and susceptibility to ROS in *per^0^* (adult) flies but information about the larval stages was lacking [21,27,28,29]. We investigated ROS levels in larvae using MitoSox Red, a fluorogenic compound targeted to the mitochondria. We detected an increased ROS burden in the CNS of *per^0^* larvae compared to *per*^+^ controls. Interestingly, MitoSox Red stained preferentially large-size cells, which is characteristic of NBs, even in *per*^+^. This agrees with previous findings that in wild-type larvae, both under normal conditions and after oxidative challenge, ROS levels and peroxidation damage are higher in NBs compared to neighbouring cells [54].

Surprisingly, using high-resolution respirometry (Oroboros oxygraph) we were unable to identify respiration defects in *per^0^* larvae. At present, we do not have an explanation for this finding, although there are some factors that may have contributed to such a result. We analyzed mitochondrial respiration in whole-body extracts, which may have masked CNS-specific differences. Additionally, because stem and progenitor cells rely more on glycolysis than OXPHOS for energy production, overall respiration in larvae is low, possibly curbing the ability to detect variation [55]. Under cold (and maybe other) conditions, larval mitochondria utilize Uncoupling Protein 4C (UCP4C) to generate heat at the expense of ATP production. In nature, this mechanism is essential for the growth of larvae at suboptimal temperatures [56]. In the laboratory, it may have contributed to concealing differences between *per^0^* and the wild type. Thus, we looked at respiration in adults and found defects in OXPHOS and electron transport in *per^0^*. Such a result aligns with evidence of augmented oxidative damage, including increased protein carbonylation, enhanced vulnerability of dopaminergic neurons, and reduced lifespan and healthspan, in *per^0^* flies (compared to *per*^+^) that were subject to oxidative stress [21,27,28]. Furthermore, our findings support previous suggestions of altered mitochondrial function in *per^0^*, which were brought forward to explain defects in lipid synthesis and upregulation of the mitochondrial stress marker 4EBP in mutant flies [29]. However, we are aware of a discordant report showing a higher metabolic rate in *per^0^* flies. The authors identified UCP4C-dependent mitochondrial uncoupling in (adult) mutants, resulting in decreased ROS production and extended longevity compared to the wild type [57]. Could the mitochondria of *per^0^* flies maintain a ‘larval state’ under their laboratory conditions? While we do not have an answer, we think that experimental differences are likely the source of our diverging results.

Overall, the available evidence suggests that in both larvae and adult *per^0^*, mitochondria are altered and (under usual laboratory conditions) generate high levels of ROS. Indeed, by buffering ROS with vitamin C (a scavenger) or by reducing their production with AOX (an alternative oxidase that ‘shortcuts’ the mitochondrial electron transport chain, diverting electrons from the production of ROS, [40]), we were able, in *per^0^*, to reduce dsDNA damage and to restore the frequency of chromosome aberrations to wild-type levels. Notably, we expressed AOX using *tim-GAL4*. This suggests that the cells important for phenotypic rescue possibly express both *per* and *tim*, which, if confirmed, qualifies them as putative clock cells.

### 4.2. PER Is Expressed in Larval Glia

In *Drosophila* the role that the circadian clock, or its components, has on development is little explored and poorly understood. In embryos, PER and TIM are expressed in many cells of the CNS from embryonic stage 12 (ES12), but only at the end of ES16 they seem to overlap in about 20 cells of the protocerebrum [13,14]. In larvae, the pattern of expression is even more uncertain. There is agreement that in each brain lobe (BL) there are nine *bona fide* clock neurons expressing rhythmic PER and TIM [33,34,47,48]. However, whether and when the other clock neurons (which are more than 75 per hemisphere in the adult) become differentiated in larvae and whether they express PER and/or TIM is not clear. For instance, by confocal microscopy, it has been shown that some dorsal clock neurons (additional to the nine referred to above) become apparent in late third-instar larvae, but they express PER at low levels and with no cycling [48]. Conversely, evidence from a spatially restricted PER-LUCIFERASE reporter has suggested that PER is rhythmically expressed in these neurons from the end of embryonic development onward [58]. These and other discrepancies may be explained by the low level of expression of the native proteins, the complex expression pattern of the reporters, and the lack of independent markers to identify clock cells reliably [33,34,47,48].

In this work, we have shown α-PER immune labelling and expression of a ‘classic’ PER reporter, SG, in larval glial cells. Kaneko and Hall (2000) identified PER expression in larval glia using a different reporter (*per-GAL4* > TAU), which provides independent confirmation of our finding [34]. Here, by combining SG with GFP expression in glia (SG, *repo-GAL4* > GFP) and by comparing the position and the morphology of the GFP-positive cells, we were able to identify some putative PER-expressing cells as cortex glia. These are large, web-like cells, born during the mid-embryonic stage, that surround and support the NBs and their lineages functioning as ‘niche’ [49]. Liu et al. (2015) did not detect PER staining in glia [48]. However, further validation of our observations comes from our manipulation experiments. By overexpressing PER (*repo-GAL4* > PER, *per^0^*) in a *per^0^* background and by mutating *per* (*repo-GAL4* > *gRNAper*, CAS9) in a *per*^+^ background, only in glia, we were able to rescue and to induce, respectively, chromosome aberrations observed in *per^0^* larvae.

### 4.3. An Altered Epigenetic Landscape in per^0^ Larvae

In *Drosophila*, H2AV is homologous to both histone variants H2AX and H2AZ found in mammals. Like H2AX, the phosphorylation of H2AV at Ser 137 (Ser 139 in H2AX) signals the presence of dsDNA breaks and leads to the recruitment of repair proteins at the break sites. Like H2AZ, the deposition of H2AV regulates transcription and chromatin structure [46]. Indeed, both H2AV and H2AZ are found at the promoter region of active genes supporting their transcription, but also in correspondence with facultative and constitutive heterochromatin contributing to their establishment and repressive function [46,59,60]. Both H2AV and H2AZ interact with insulator proteins and regulate their deposition across the genome [61,62]. Insulators mediate the 3D organisation of chromatin by promoting the formation of topologically associating domains (TADs) that establish ‘locally regulated areas’ of gene expression [63,64]. The phosphorylation of H2AV to γ-H2AV leads to the activation of PARP1, an enzyme that transfers ADP-ribose units from NAD^+^ to target proteins. Such a process promotes the opening of chromatin by de-stabilizing pre-existing protein complexes, which facilitates transcription and genotoxic stress responses [51]. These findings suggest that we may find epigenetic dysregulation in *per^0^* such as a reduction in chromatin compaction. Indeed, in mammalian fibroblasts, a triple *Per1-3* knock-out (that removes the three homologous *Per* genes) causes reduced deposition of H2AZ, greater genome accessibility and persistent DNA damage [10]. Thus, we overexpressed in all putative clock cells of the larva (using *tim-GAL4*) SU(VAR)3-9 and HP1, which are involved in the formation and maintenance of H3K9me3, a fundamental component of constitutive heterochromatin [53]. Both manipulations rescued the chromosome aberrations that were otherwise observed in *per^0^* mutants, suggesting that PER is required for the correct establishment or maintenance of chromatin architecture.

### 4.4. Mechanistic Implications: ROS, ‘Neuroblast Sparing’ and PER

During development, metabolic stressors, such as insufficient nutrients or oxygen, can affect the growth of organs and tissues. However, the growth of the brain is largely spared. This phenomenon, known as ‘brain sparing’, takes place in organisms as diverse as mammals and insects [65]. Several stressors result in an increase in ROS, which can cause oxidative damage to lipids, proteins and nucleic acids. In particular, lipid oxidation can lead to the formation of lipid hydroperoxides that decompose in reactive chemical species triggering a chain reaction [66]. Hence, neuronal stem cells, which are critical for the development of the CNS, must be ‘spared’ from the deleterious effects of ROS. Evidence has shown that ROS (which are higher in NBs) stimulate the production of lipid droplets (LDs) in glia, especially cortex glia that envelop the neuroblast lineages, while neither NBs nor neurons produce them [54,67]. Moreover, the production of LDs confers to glia the ability to protect the neuroblast lineages (and themselves) from ROS [54]. The detailed molecular mechanisms regulating this non-cell autonomous protection process are currently unknown. However, it strongly resembles the lipid metabolic cycle between adult neurons and glia that protects both cell types from oxidative damage and regulates sleep [68].

**Figure 7:**
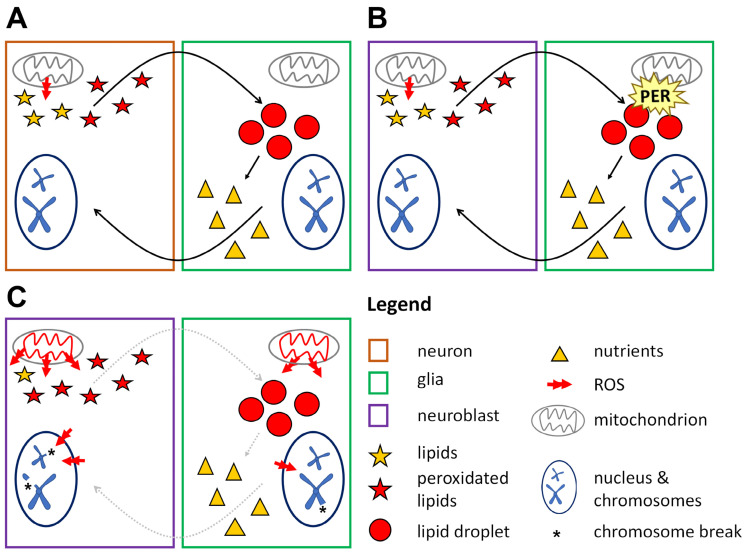
Lipid droplets in glia have a protective antioxidant role. (**A**) Glia–neuron lipid cycle in adult flies. During the day neurons are active and produce ROS that trigger the synthesis of lipids. These become peroxidated by ROS and are exported to glia. In glia, peroxidated lipids are sequestered into LDs (preventing chain reactions and further damage) during the day (when flies are awake) and then are broken down and re-exported at night (when flies sleep). Thus, glia detoxify incoming peroxidated lipids and convert them into energy-producing molecules that are exported back to neurons [67]. (**B**) Hypothetical glia–NB cycle in wild-type larvae. A metabolic cycle (like the one described above) protects the cells from oxidative stress and sustains their metabolic needs. PER expression in glia is necessary to maintain mitochondrial function and LDs turnover (**C**) Hypothetical glia–NB interactions in *per^0^* larvae. Absence of PER breaks the protective lipid cycle, resulting in high levels of ROS, mitochondrial dysfunction, changes in chromatin structure and chromosome damage.

We speculate that PER may be involved in a protective metabolic cycle occurring between glia and NBs (Figure 7). Our data show that ROS are at the core of chromosome aberrations in dividing neuronal precursors in *per^0^* mutants and that PER expression limited to glia is necessary and sufficient to prevent chromosome defects. Further work will be required to establish whether lack of PER affects primarily the production and turnover of LDs in the glia and then the mitochondrial respiratory chain, or vice versa. Regardless, the dysfunction triggers abnormal levels of ROS, causing, we suggest, chromatin decompaction through the persistent activation of the DNA damage response. Such a change in chromatin conformation enables further damage, resulting in chromosome aberrations. Indeed, the expression of enzymes involved in the establishment and maintenance of (compact) constitutive heterochromatin rescues the phenotype. Future investigations will tackle the molecular mechanisms and will be extended to the chromatin of neurons and glia in (adult) *per^0^* flies.

In summary, our work shows how a fundamental constituent of the circadian clock, possibly the clock itself, can influence metabolism and chromatin structure to regulate development, which perhaps points to a fundamental function of the clock that goes beyond rhythmicity.

## Data Availability

This work did not generate novel materials or tools. Data and materials used are available from the corresponding authors upon request.

## Supplementary Materials

The following supporting information can be downloaded at: https://www.mdpi.com/article/10.3390/cells13231944/s1, Figure S1: Schematic representation of the central nervous system (CNS) of *Drosophila* larvae; Figure S2: High resolution respirometry does not identify differences between *per*^+^ and *per^0^* in third-instar larvae; Figure S3: ROS buffering decreases DNA damage in *per^0^*.
