# Supplementary material for: Neuronal Progenitors Suffer Genotoxic Stress in the Drosophila Clock Mutant per0
Source: Cells. 2024 Nov 23;13(23):1944. doi: 10.3390/cells13231944 (PMC11640223; doi:10.3390/cells13231944)
Supplement: Supplementary file 1 [file cells-13-01944-s001.zip › cells-3274697-supplementary.pdf]

Supplementary Information for

Neuronal progenitors suffer genotoxic stress in the *Drosophila* clock mutant *per*[0]

Nunzia Colonna Romano[1,2,†],Marcella Marchetti[1,†], Anna Marangoni[1], Laura Leo[1,3], Diletta Retrosi[1,2], Ezio Rosato*[2,1], & Laura Fanti*[1,4]

Corresponding authors: Ezio Rosato: er6@leicester.ac.uk
Laura Fanti: laura.fanti@uniroma1.it

This PDF file includes:

Figs. S1, S2, S3

Captions for Figures S1, S2, S3

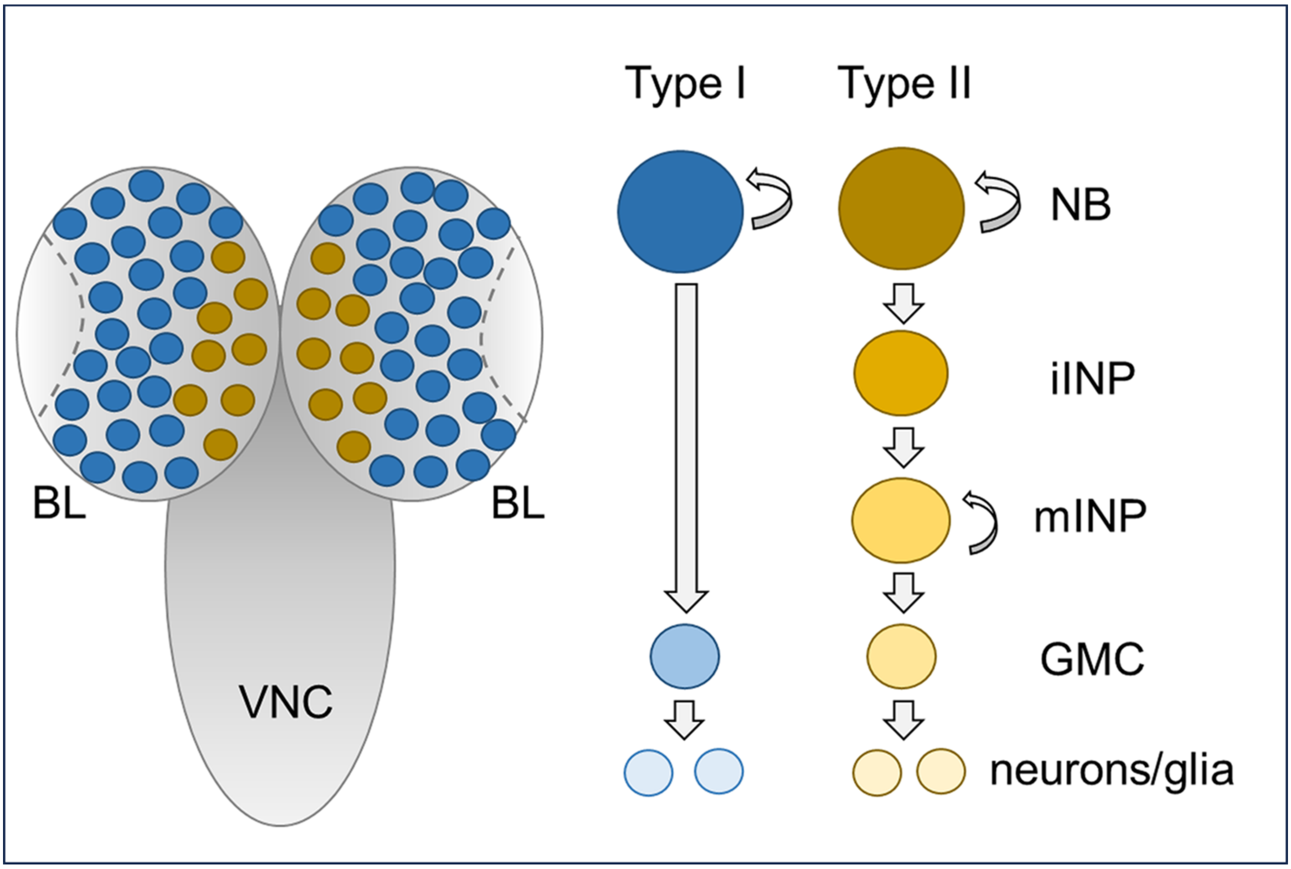

Figure S1. Schematic representation of the central nervous system (CNS) of *Drosophila* larvae. Distribution of type I (blue) and type II (gold) neuroblasts in the brain lobes. In the ventral cord, only type I neuroblasts are present (not shown). BL: brain lobe; VNC: ventral nerve cord; NB: neuroblast; iINP: immature intermediate neural progenitor; mINP: mature intermediate neural progenitor; GMC: ganglion mother cell.

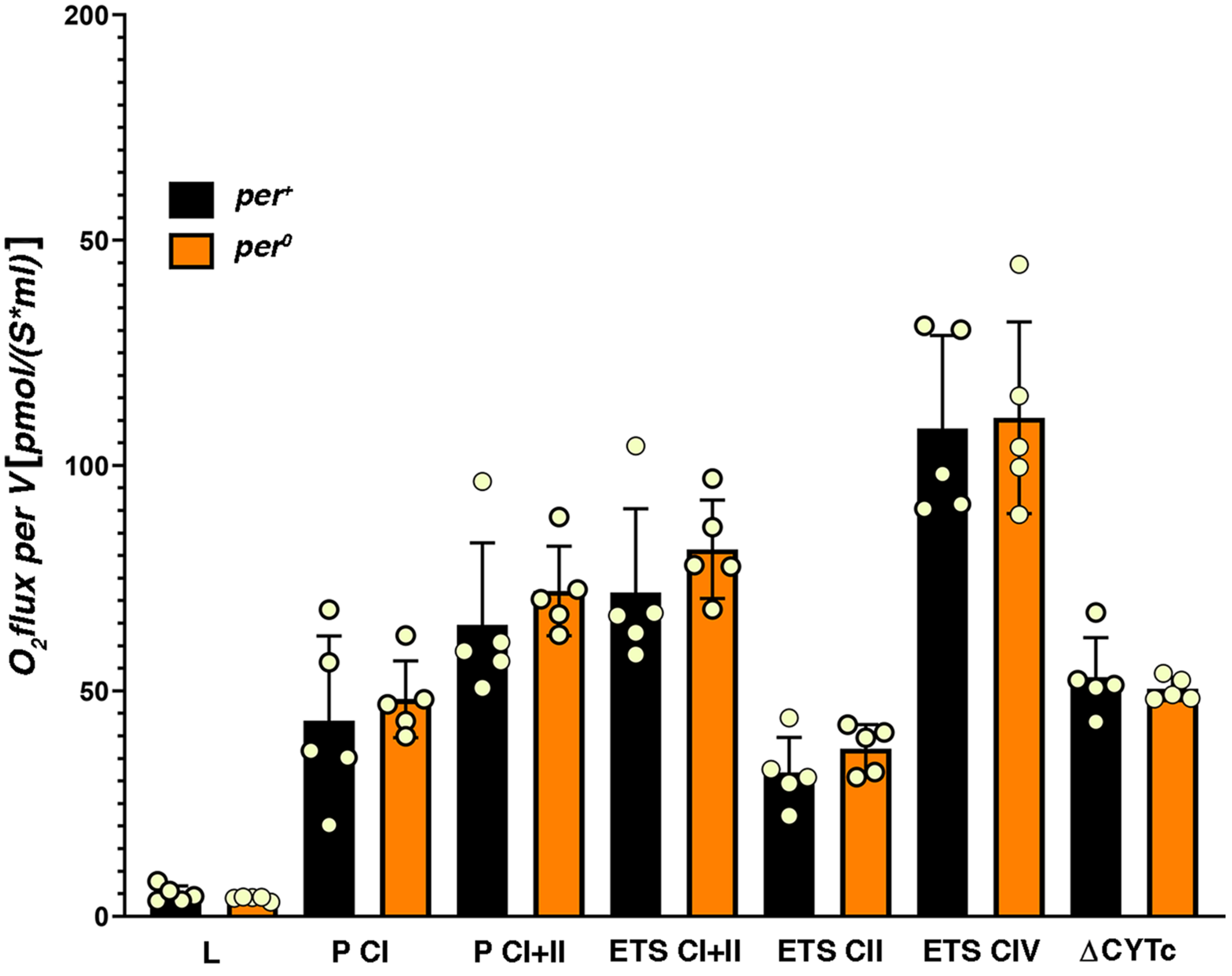

Figure S2. High resolution respirometry does not identify differences between *per*$^{+}$ and *per*^{0} in 3rd instar larvae. L, mitochondrial leak (Mann-Whitney, P=0.421). P CI, OXPHOS capacity of complex I (Mann-Whitney, P=0.548). P CI + II, OXPHOS capacity of complex I plus II (Mann-Whitney, P=0.151). ETS CI + II, electron transport capacity through complex I plus II (Mann-Whitney, P=0.151). ETS CII, electron transport capacity through complex II (Mann-Whitney, P=0.310). ETS CIV, electron transport capacity through complex IV (Mann-Whitney, P=0.841). ΔCYTc, increase in ETS CIV when excess cytochrome c is added to the respiration buffer (measure of mitochondria integrity, Mann-Whitney, P=0.691). Points show individual samples. Error bars = SD. ZT=1. All samples were males obtained by reciprocal crossing (♀ *CS* x ♂ *per*^{0} and *vice versa*).

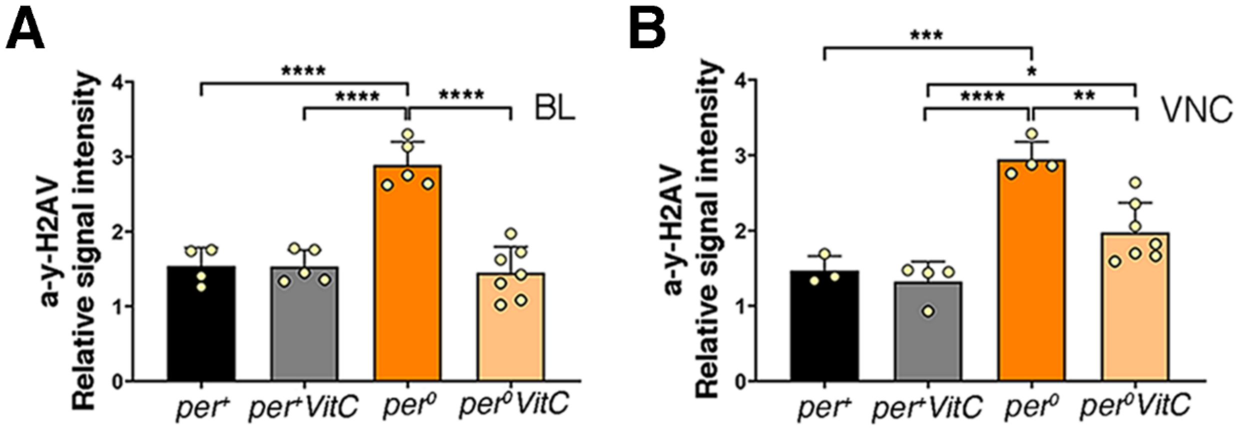

Figure S3. ROS buffering decreases DNA damage in *per⁰*. Quantification of anti-γ-H2AV immune fluorescence intensity [relative signal intensity = (signal-background)/background] in whole mount CNS from 3rd instar *per*+ and *per⁰* male larvae obtained by reciprocal crossing (♀ *CS* x ♂ *per*⁰ and *vice versa*). Larvae raised on standard medium were compared to larvae treated with vitamin C (VitC, 40 mM in standard medium) from embryo. This experiment was carried out independently from the one shown in Fig 3B and was imaged on a different microscope. Normal distribution of data was confirmed with the Shapiro-Wilk test. A) BL (one per individual). Two-way ANOVA, Genotype ($F_{1,\ 14}$=21.03, p=0.0004), VitC treatment ($F_{1,\ 14}$=40.05, p<0.0001), Genotype x VitC treatment ($F_{1,\ 14}$=39.53, p<0.0001). Tukey's multiple comparisons test, *per*+ *vs*. *per⁰*, ****p<0.0001; *per*+ VitC *vs*. *per⁰*, ****p<0.0001; *per*⁰ *vs*. *per⁰* VitC, ****p<0.0001. B) VNC. Two-way ANOVA, Genotype ($F_{1,\ 14}$=46.94, p<0.0001), VitC treatment ($F_{1,\ 14}$=12.97, p=0.0029), Genotype x VitC treatment ($F_{1,\ 14}$=7.040, p=0.0189). Tukey's multiple comparisons test, *per*+ *vs*. *per⁰*, ***p=0.0001; *per*+ VitC *vs*. *per⁰*, ****p<0.0001; *per*+ VitC *vs*. *per⁰*, *p=0.0236; *per*⁰ *vs*. *per⁰* VitC, **p=0.0011. Points show individual samples. Error bars = SD. ZT=1.
